# Severity and Endurance in Eating Disorders: An Exploration of a Clinical Sample From Chile

**DOI:** 10.3389/fpsyt.2020.00869

**Published:** 2020-08-28

**Authors:** Marcela M. Dapelo, Aurora A. Gil, Lucas Lacalle, Melina Vogel

**Affiliations:** ^1^Center for Studies in Eating Behavior, School of Psychology, Universidad Adolfo Ibáñez, Santiago, Chile; ^2^Eating Disorders Unit, Psychiatry Department, Faculty of Medicine, Pontificia Universidad Católica, Santiago, Chile

**Keywords:** severe and enduring eating disorders, chronicity, access to treatment, eating disorder complications, clinical impairment, quality of life

## Abstract

**Introduction:**

One in five patients with eating disorders (EDs) may take a lasting course. It has been proposed that this enduring group exhibits increased severity, such as low treatment response, severe symptomatology, and poor quality of life. However, there is no consensus defining this group. Moreover, most of the literature comes from high-income societies and may not apply to developing countries.

**Aims:**

This study aimed to evaluate the association between endurance (length of ED) and severity (previous treatments, hospitalizations, medical complications, symptomatology and clinical impairment) in individuals with EDs from Chile. In addition, it aimed to explore the association between endurance and delays seeking specialized treatment.

**Methods:**

Forty-one women with EDs (16 with anorexia nervosa, 11 with bulimia nervosa, 10 with binge eating disorder and 4 with other specified EDs) completed a social-demographic survey, the Eating Disorders Examination Questionnaire, and the Clinical Impairment Assessment. Also, Body Mass Index, length of ED, and complications were retrieved from participants’ medical records. Spearman correlation coefficient and linear regression were used to explore the association between length of ED and measures of severity and treatment seeking behavior.

**Results:**

There was no significant association between endurance (length of ED) and measures of severity. There was a significant association between length of ED and delays seeking specialized treatment (r_s_=0.72; p<0.01). Regression indicated that for each month in delay visiting a specialized ED treatment team, the ED duration increased by 0.87 months (F(1,38)=75.93; p<0.01; R^2^= 0.66).

**Discussion:**

The findings suggest that in developing countries, where specialized treatment access is not widespread, defining SEED solely by the length of illness may not be clinically significant, and other criteria (e.g., timely access to evidence-based treatments) should be considered.

## Introduction

Eating disorders (EDs) can take a lasting course over time, which is usually associated with higher resistance to usual treatments and a more significant overall impact on individuals who suffer from it. It has been reported that 20% of cases with anorexia nervosa (AN) and 23% of patients with bulimia nervosa (BN) would take this course (1, 2).

There is growing interest in scientific literature to define chronicity in EDs. The first approach to the concept of severe and enduring EDs (SEED) appeared in 1999 when the “severe and enduring” descriptor for mental illness (SEMI) was published by the National Service Framework of the United Kingdom to direct resources to patients suffering from long term severe disorders. It was defined as: “people with recurrent or severe and enduring mental illness, for example, schizophrenia, bipolar affective disorder or organic mental disorder, severe anxiety disorder or severe eating disorder, have complex needs which may require the continuing care of specialist mental health services working effectively with other agencies” (3). Later, it was proposed for EDs, suggesting that a mental disorder that has a mortality rate several times higher than schizophrenia, should not be excluded from disorders carrying the severe and enduring label. Robinson described the term severe and enduring eating disorder (SEED) for patients with severe and prolonged disease in bulimia nervosa with the suffix of BN. and those with anorexia nervosa, SEED-AN (4, 5). However, there is no consensus on how severe or long-lasting the disease should be to be labeled as SEED.

In his systematic review and critical analysis of studies concerning lasting forms of AN, Broomfield found that the duration of the disease was the most common criterion used to define what we understand for SEED (used in 84% of the studies analyzed), with a presentation of an AN for 7 years as the most commonly used duration criterion. The second most common criterion was the number of failed treatment attempts, but most articles did not adequately develop this point. Other criteria that defined severe and prolonged AN were body mass index, the persistence of behavioral or cognitive patterns of AN, alteration of various areas of life, low motivation for recovery, and presence of severe symptoms (6).

Hay and Touyz propose an alternative definition of SEED, using the following criteria: (a) a persistent state of dietary restriction, underweight, and overvaluation of weight/shape with functional impairment, (b) duration of > 3 years of AN and (c) exposure to at least two evidence-based treatments appropriately delivered together with a diagnostic assessment and formulations that incorporates an assessment of the person’s eating disorder health literacy and stage of change (7).

Even though most of the research has focused on severe and enduring forms of AN, lasting courses are common in other EDs, such as BN (2). In addition, ED diagnosis is highly unstable, and between 27% and 34% of individuals with AN may crossover to BN within 6 to 7 years (8, 9), and between 15% and 31% of AN may switch to ED not otherwise specified (9). Thus, a more general approach that conceptualizes EDs’ course in terms of stages has been developed, proposing that not only AN, but also BN’s course (and potentially BED) could be mapped into the following stages: high risk, prodromal, full syndrome, and severe and enduring (10).

The SEED concept assumes that EDs with an enduring course are associated with increased severity (11). In fact, research shows that physical complications are often associated with long-lasting EDs. Almost every organ system can be affected secondary to chronic malnutrition, bingeing, and/or purging behaviors, as well as those individuals with marked overweight or weight fluctuations, with frequent musculoskeletal, endocrine/reproductive, cardiovascular, hematological, gastrointestinal, renal, neurologic, and hydro-electrolyte disturbances, among others (12–15). There is some evidence that this group of patients is more likely to resist change to their ED, though they might be willing to reduce the immediate physical risk. Therefore, the approach to the treatment is crucial, addressing physical needs in the first place, providing compassionate bio (nutritional) psychosocial support (16).

It is common for people with EDs to experience a significant impact of the disorder on their life and close environment (17). In the case of SEED, studies show a more considerable disturbance in the quality of life. Virtually all personal fields are affected, such as physical and psychological integrity, family and social networks, functional level, personal and family economics (11, 18). It is common to observe that there is a variable degree of disability attributable to the disorder and sometimes also to the treatments that have been attempted without success (18).

Most of the literature described above comes from studies carried out in high-income, western societies. However, there is a lack of information about long-lasting forms of EDs coming from other parts of the world. Thus, it is unclear if the current state of the art applies to other societies, such as Latin America. In this context, the study aimed to evaluate the association between endurance, measured as the length of the ED, and clinical characteristics that are usually considered measures of severity, such as the number of previous treatments and hospitalizations for the ED, number of medical complications, ED symptoms and clinical impairment in individuals with ED from Chile. In addition, we explored the association between length of the ED and delays seeking specialized treatment. To our knowledge, this is the first study exploring SEED in the Latino American population.

## Materials and Methods

### Design and Participants

The current research is a cross-sectional exploratory design study of patients who were referred to an Eating Disorder Unit in Santiago, Chile. Participants were recruited from the outpatient specialist eating disorder treatment program. As a part of the routine care, all patients were initially screened by a trained clinician to determine ED symptomatology according to the DSM-5. After completing this clinical interview, they were asked if they wanted to participate in the study. Those who expressed interest were contacted by a researcher who explained the research further and sought informed consent. Inclusion criteria were being 18 years old or older with a current ED diagnosis. Those who had active substance abuse (information obtained in the clinical interview) were excluded.

### Measures

The study was conducted using a set of three self-report measures completed by the participants under the supervision of a researcher (Social-Demographic Survey, EDE-Q, and CIA). Weight, height, and body mass index (BMI= weight (kg)/[height (m^2^)]) were evaluated by one of the authors under a nutritional assessment protocol, at the time of diagnosis. These anthropometric measurements were obtained on calibrated scales with the participants wearing underwear. Information about length of ED and medical complications (specifically, musculoskeletal, cardiovascular, endocrine/reproductive, gastrointestinal, dental, hydroelectrolytic, hematological, renal, and/or vitamin deficiencies) were retrieved by hand from medical records.

#### Social-Demographic Survey

Data on disease information, marital status, occupation, and educational level were assessed in an investigator-designed demographic survey.

#### EDE-Q

Eating Disorders Examination Questionnaire [EDE-Q v.6; (19, 20)] The EDE-Q 6th version is a 28-items measure assessing cognitive and behavioral features of ED. It has four subscales (dietary restraint, eating concern, weight concern, and shape concern) and a global score. The Spanish version adapted for Chile was used (Gaete, Lopez, manuscript in preparation). In the study sample, Cronbach’s alpha for the global score = 0.95.

#### CIA

Clinical Impairment Assessment [CIA 3.0; (21)]: The CIA is a 16-item self-report measure designed to assess the ED’s impact on psychosocial functioning, specifically on personal, cognitive, and social domains. The CIA has shown good reliability and validity (21). The Spanish version has been adapted to the Chilean population (22, 23). In the current study sample, Cronbach’s alpha = 0.95.

### Procedure

Patients who expressed interest and met the criteria to participate in the study were contacted by a team researcher and given details of the study. Participants who agreed to take part signed informed consent and completed the study questionnaires in person, in front of one of the researchers. Participants did not receive compensation. The Research Ethics Committee of the Faculty of Medicine of the Catholic University of Chile approved the study.

### Statistical Analysis

Descriptive statistics were calculated to characterize the sample regarding demographic and clinical characteristics. Data distributions were inspected using histograms and Kolmogorov-Smirnov test of normality. Since none of the clinical variables exhibited a normal distribution, Spearman rho correlation was used to assess the association between length of ED and clinical variables (Previous treatments, hospitalizations, medical complications, delay visiting specialized ED treatment team, EDE-Q and CIA global scores). Statistical significance was adjusted for multiple testing, according to Bonferroni. Variables that were significantly correlated to length of ED were entered in a linear regression model to assess the extent to which these variables explained variations in length of ED. Bootstrapping based on 1000 bootstrap samples was carried out to obtain 95% confidence intervals for B in the linear regression. All analyses were conducted using SPSS v.26.

## Results

Forty-nine individuals with EDs joined the study. In order to reduce heterogeneity, it was decided to remove male participants and those with an uncommon diagnosis. Thus, data from 4 males (3 with other specified ED and 1 with binge eating disorders), 2 women with avoidant restrictive food intake disorder, and 2 women with unspecified ED were removed from the analyses, yielding a final sample of 41 female participants.

In the final sample (n = 41), participants’ age ranged from 18 to 51 years (mean = 28.93; SD = 8.31). Most of the participants were single (n = 35; 85.40%), had university-level studies (n = 30; 73.20%), and were not working by the time of the assessment (n = 21; 51.20%). Participants’ diagnosis were: anorexia nervosa (n = 16; 39.00%), bulimia nervosa (n = 11; 26.80%), binge eating disorder (n = 10; 24.40%), and other specified ED (n = 4; 9.80%).

Participants’ clinical characteristics are shown in **Table 1**. Length of the ED ranged from 1 to 26 years (specifically, 12.00 to 317.00 months). On average, participants first visited a specialized ED treatment team 7 years after experiencing ED symptoms for the first time (86.40 months), had 2 previous specialized treatments, and 0.68 hospitalizations for the ED. At the time of the assessment, participants showed an average of 1.37 medical complications (i.e., musculoskeletal, cardiovascular, endocrine/reproductive, gastrointestinal, dental, hydroelectrolytic, hematological, renal, and/or vitamin deficiencies), and obtained an average score of 3.66 on the EDE-Q and of 30.49 on CIA.

**Table 1:** Participants’ clinical characteristics.

	Min–Max	Mean (SD)	Median (IR)
Length of ED in months (n = 41)	12–317	119.27 (93.15)	77.00 (174)
Delay in visiting first specialized ED treatment team in months (n = 40)	0–306	86.40 (88.23)	51.50 (114.00)
Number of previous specialized treatments (n = 35)	0–9	2.00 (2.02)	1.00 (2.00)
Number of hospitalizations for ED (n = 38)	0–5	0.68 (1.17)	0.00 (1.00)
Number of medical complications (n = 41)	0–6	1.37 (1.67)	1.00 (2.00)
EDE-Q Global score (n = 41)	0–6	3.66 (1.48)	4.01 (2.00)
CIA Global score (n = 41)	0–46	30.49 (12.29)	34.00 (18.00)

Min, minimum; max, maximum; SD, standard deviation; IR, interquartile range; n, number of participants with valid data.

A significant direct association was found between the length of the ED and the delay in visiting for the first time a specialized ED treatment team (r_s_ = 0.72; p < 0.01; adjusted p < 0.01). The length of the ED was not significantly associated with the number of previous treatments for ED (r_s_ = 0.20; p = 0.24; adjusted p = 1.00), number of hospitalizations for ED (r_s_ = 0.27; p = 0.10; adjusted p = 0.58), number of medical complications (r_s_ = 0.07; p = 0.65; adjusted p = 1.00), EDE-Q global score (r_s_ = 0.21; p = 0.20; adjusted p = 1.00), nor with the CIA global score (r_s_ = −0.05; p = 0.75; adjusted p = 1.00).

Since the only variable showing a significant association with the length of the ED was the delay in visiting for the first time a specialized ED treatment team, only the latter was used as a predictor in the regression analysis. Results from the linear regression indicated that the delay in visiting for the first time a specialized ED treatment team significantly predicted variations in length of the ED (F(1,38) = 75.93; p < 0.01; R^2^ = 0.66). Findings showed that the length of the ED increased by 0.87 months for every 1 month of delay in consulting to an ED specialized team (B = 0.87; 95% CI = 0.73–1.01; β = 0.82; t = 8.66; p < 0.01)^^1^^.

## Discussion

By definition, individuals with SEED are thought as having more severe symptoms (11). However, most of the literature comes from studies carried out in high-income western countries, and it is unknown if the profile described for SEED applies to developing societies. In this context, the present study aimed to evaluate the association between endurance, measured as the length of the ED, and clinical characteristics that are usually used to describe severity in a sample of individuals with EDs from Chile. Contrary to our expectations, the study findings showed no significant association between length of the ED and number of previous ED treatments, number of hospitalizations for EDs, number of medical complications, ED symptomatology (assessed through the EDE-Q), and functional impairment (assessed through the CIA).

The findings are not in line with previous research. SEED patients are usually characterized as having multiple previous treatment failures, more physical complications, and poor quality of life (24). In addition, there is evidence of higher scores in global EDE-Q in SE-AN (defined as an AN lasting longer than 3 years), compared to a presentation of fewer than 3 years [Giardini, cited in (25)]. Considering that the most common criterion used to define SEED is based on illness duration, a significant association between length of ED and these variables was expected.

One possible explanation for the discrepant findings may relate to the study participants. Most of the literature in SEED has been carried out in AN. However, our sample includes participants with AN, BN, BED, and other specified ED. Even though we explored the results for AN and were similar to the findings exhibited for the whole sample, this analysis was underpowered, and therefore, it is not possible to be certain that the transdiagnostic approach used in the study did not affect the findings.

There is current discussion regarding the best approach to define SEED, and even though most studies use ED duration as a criterion, there is no consensus regarding a specific threshold to qualify as SEED, and regarding which other criteria should be used in addition to illness length (6). Moreover, the discussion has been largely based on experts’ opinion, and empirically driven criteria are needed (26). In fact, findings from a study using empirical techniques to classify SEED suggest that the severity and endurance of the disease may not necessarily be correlated, and that other clinical aspects are essential to consider in the definition (27). This is in line with our findings, highlighting the relevance of avoiding relying solely on ED duration to define SEED, and avoiding assuming its link to severity.

In our study, the only variable that exhibited a significant association to length of the ED was the delay in visiting a specialized ED treatment team. Regression analysis indicated that for each month of delay consulting a specialized team for the first time, since first experiencing symptoms, the length of the ED increases by 0.87 months. This delay obtaining specialized treatment accounted for 66% of the variability in ED duration. It has been described in the literature a delay in specialized treatment for EDs (28). In countries where access to specialists is not widespread, primary care is carried out by non-specialists who may not see as necessary to ask about eating patterns in children, adolescents, or adult patients. In Chile, ED incidence is relatively low compared to other mental health disturbances (29), which has limited the allocation of resources for early detection and treatment. The Chilean health system is mixed, public, and private, with the public system being the one that takes care of the low income, senior citizens, and with a more significant disease burden population. Mental health service provision in Chile still lacks enough resources for addressing high prevalence psychiatric problems (30). EDs have not been adequately visualized as prevalent, severe, and capable of generating disability in young people, mostly because of the lack of studies of prevalence, course, and characterization of the population suffering from these disorders in our country. In this scenario, people with EDs may find it difficult to access specialized treatment and may experience symptoms for long periods. However, as suggested by the study findings, this group of individuals may not represent a severe group and may not need a specifically tailored treatment.

These results may imply that, at least for countries in which specialized treatment for EDs is not widespread, defining SEED based only on ED duration is not adequate. Other criteria, such as the proposed by Hay and Touyz (7), which include the exposure to at least two evidence-based treatments appropriately delivered, may have more clinical utility.

### Limitations

The findings of the study must be taken in the context of its limitations. First, the sample size is small. In addition, even though we removed data from uncommon diagnoses to reduce heterogeneity, participants’ diagnoses were still diverse. In particular, it is unclear if the severe and enduring label should be applied to individuals with BED (31), so their inclusion could be questioned. Future studies may aim at having larger sample sizes, and at exploring the long-lasting ED patients’ response to evidence-based treatments longitudinally.

## Conclusion

In conclusion, the study found no evidence for an association between endurance, measured as the length of the ED, and measures of severity, such as the number of previous treatments and hospitalizations for the ED, number of medical complications, ED symptoms, and clinical impairment. The variation in ED duration was better accounted for by the delay in obtaining specialized ED treatment since the onset of symptoms. In a context where mental health treatment is not widely accessible, the finding highlights the need not to assume that endurance and severity are always linked, and to complement the duration criterion with others, such as previous exposure to evidence-based treatment, in order to identify SEED patients.

## Data Availability Statement

The datasets generated for this study are available on request to the corresponding author.

## Ethics Statement

The study involved human participants and was reviewed and approved by Research Ethics Committee of the Faculty of Medicine of the Catholic University of Chile. The patients/participants provided their written informed consent to participate in this study.

## Author Contributions

MD, AG, and MV conceived the study. MV obtained ethics approval. MV, AG, and LL recruited participants and obtained data. LL organized the database. MD performed statistical analyses. All authors contributed to the article and approved the submitted version.

## Conflict of Interest

The authors declare that the research was conducted in the absence of any commercial or financial relationships that could be construed as a potential conflict of interest.

## Footnotes

1 To explore if the results had been distorted by the inclusion of data from participants with different ED diagnoses, all analyses were repeated in the subgroup with AN (n=16), yielding the same findings (data available upon request).
